# Current research on the HIF-2α-EPO–Hb axis in hypoxic environments: from molecular mechanisms to clinical

**DOI:** 10.3389/fmed.2026.1780669

**Published:** 2026-02-23

**Authors:** Qiaochu Zheng, Yinglan Li, Jimei Li

**Affiliations:** 1 Qinghai University, Xining, China; 2 Qinghai Provincial People's Hospital, Xining, China; 3 Qinghai Clinical Research Center for High Altitude Diseases, Xining, China

**Keywords:** chronic disease, erythropoietin, hemoglobin, HIF-2α, high-altitude adaptation, hypoxic environment

## Abstract

Hypoxic environments modulate downstream gene expression via activation of the hypoxia-inducible factor (HIF) signaling pathway, which facilitates adaptive responses to low oxygen. Under hypoxic conditions, HIF-2α is stabilized and translocated to the nucleus, where it binds to hypoxia-response elements and upregulates transcription of the erythropoietin (EPO) gene. Increased EPO stimulates red blood cell production in the bone marrow, raising hemoglobin (Hb) levels to improve oxygen transport. This review examines the principal regulatory pathway, the Hypoxia-Inducible Factor-2α-Erythropoietin–Hemoglobin axis (HIF-2α-EPO-Hb), which governs physiological and pathological responses to hypoxia. The HIF-2α-EPO-Hb axis dynamically regulates erythropoietin and hemoglobin production in response to fluctuations in oxygen levels, maintaining systemic oxygen homeostasis. This pathway is involved in both physiological processes, such as high-altitude adaptation, and pathological conditions. Disruption of this axis leads to anemia associated with chronic kidney disease, while excessive activation contributes to high-altitude polycythemia and tumor progression, including renal cell carcinoma. The complex regulatory networks of this axis across diverse tissue microenvironments and disease states remain incompletely characterized. Targeted interventions, such as hypoxia-inducible factor-prolyl hydroxylase inhibitors and hypoxia-inducible factor-2α inhibitors, face significant challenges in tissue selectivity, long-term safety, and efficacy prediction. This review elucidates the molecular regulatory mechanisms of the HIF-2α-EPO-Hb axis, delineates its dysregulation in chronic hypoxic diseases and tumorigenesis, and evaluates current research progress and clinical limitations of related therapies. The discussion provides theoretical foundations and future perspectives for mechanistic research and clinical intervention in relevant diseases.

## Introduction

Hypoxia, defined as an insufficient oxygen supply for normal physiological function, can be categorized as acute or chronic. Acute hypoxia arises from sudden high-altitude exposure or traumatic hypoxemia, whereas chronic hypoxia is observed in conditions such as chronic obstructive pulmonary disease (COPD), obstructive sleep apnea-hypopnea syndrome, chronic kidney disease (CKD), and prolonged high-altitude residence. Notably, hypoxia is a common feature of various pathological states, with the hypoxia-inducible factor (HIF) pathway serving as the central molecular mechanism for cellular detection and adaptation. This pathway is integral to biological processes, such as angiogenesis, energy metabolism, cell proliferation, survival, and inflammatory responses. Central to this system is the HIF transcription factor, which comprises hypoxia-inducible factor-α (HIF-α) and hypoxia-inducible factor-β (HIF-β) subunits. In particular, the HIF-α subunit includes factor-1α (HIF-1α), factor-2α (HIF-2α), and factor-3α (HIF-3α), with HIF-1α and HIF-2α being the most extensively studied (1, 2). Their distinct roles in response to oxygen fluctuation underpin various adaptive processes. Specifically, HIF-1α primarily drives glycolysis and the transcription of angiogenesis-related genes during short-term hypoxia, while HIF-2α is involved in slower adaptive responses and exhibits distinct tissue expression patterns. Importantly, HIF-2α serves as a key transcription factor for erythropoiesis and oxygen homeostasis (2, 3). In contrast to HIF-1α, HIF-2α directly regulates erythropoietin (EPO) transcription, thereby promoting hemoglobin (Hb) synthesis and forming the hypoxia-inducible factor-2α-erythropoietin–hemoglobin axis (HIF-2α-EPO-Hb). The precise regulation of this axis supports physiological adaptation to hypoxia, whereas its dysregulation is associated with conditions such as chronic renal anemia, high-altitude polycythemia, and certain EPO-dependent malignancies. Understanding these regulatory mechanisms is therefore critical for elucidating disease pathophysiology and informing the development of targeted therapies. Aligning with these objectives, this review first examines the normal regulatory network of the HIF-2α-EPO-Hb axis, then explores its dysregulation and potential therapeutic strategies in related diseases.

## 1 Molecular regulatory mechanisms of the HIF-2α-EPO–Hb axis

The function of the HIF-2α-EPO–Hb axis relies on its precise molecular control pathways. Therefore, this chapter will proceed along the physiological signaling direction of this axis. We will first meticulously explain how HIF-2α achieves precise regulation of EPO, followed by an analysis of how EPO in turn regulates erythropoiesis and Hb synthesis. This approach will comprehensively delineate the physiological mechanisms underlying this axis, which also serves as the foundation for understanding its dysregulation in various diseases discussed in subsequent chapters.

### 1.1 Regulation of EPO by HIF-2α

#### 1.1.1 Transcriptional regulation of the EPO gene

EPO, the core cytokine for erythropoiesis, exhibits gene expression that is precisely controlled by HIF-2α within a specific temporal window. Within the human genome, the3' enhancer region of the EPO gene contains a conserved hypoxia response element (HRE) that specifically binds to the HIF-2α/aryl hydrocarbon receptor nuclear translocator (ARNT) heterodimer. Unlike HIF-1α, which broadly activates transcription during acute hypoxia, HIF-2α exhibits a higher affinity for this HRE, enabling tissue-specific expression in organs like the kidney and liver, making it the dominant subtype governing EPO transcription (4–6). In the kidney, renal cortical interstitial fibroblasts are the primary site of EPO production under hypoxic stress. The regulatory mechanism involves a complex oxygen-sensing system: when local oxygen partial pressure falls below approximately 35 mmHg, Proline hydroxylase (PHD) inactivation, allowing HIF-2α protein stabilization and transcriptional activation of the EPO enhancer (7–9). Hepatocytes and hepatic stellate cells can also synthesize EPO, serving as an important extra-renal source in patients with renal failure and during fetal development (10–13). Due to differences in oxygen sensor distribution within their respective microenvironments, the kidney is more sensitive to systemic hypoxia, while the liver is more responsive to local hypoxia (14, 15).

#### 1.1.2 Signal transduction networks

**Figure 1:**
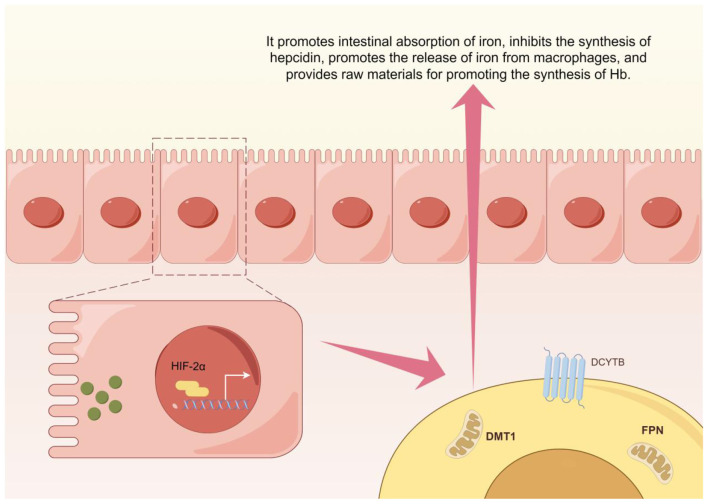
HIF-2α iron metabolism regulation diagram This schematic diagram summarizes the central role of HIF-2α in regulating systemic iron metabolism. Under hypoxic or related pathological conditions, activated HIF-2α enhances dietary iron absorption by upregulating the expression of intestinal iron transporter DMT1 and iron reductase Dcytb. Concurrently, it suppresses hepatic hepcidin synthesis and promotes iron release from macrophages, collectively elevating plasma iron availability to supply essential raw materials for hemoglobin synthesis. This figure was created using the Figdraw platform.

The regulation of EPO by HIF-2α is not an isolated pathway but operates within an interconnected signaling network. For instance, within the inflammatory signaling pathway, TNF-α can reduce HIF-2α transcriptional activity via the nuclear factor kappa-B (NF-κB) pathway, contributing to EPO resistance in the microinflammatory state of CKD (16). Regarding iron metabolism, HIF-2α directly upregulates duodenal cytochrome b (Dcytb), divalent metal transporter 1 (DMT1), and ferroportin (FPN) to enhance intestinal iron absorption. Simultaneously, it suppresses hepatic hepcidin synthesis and promotes iron release from macrophages, providing the raw materials for Hb synthesis (17, 18) (Figure 1). Another example involves ubiquitin-specific protease 7 (USP7), which is highly expressed in renal clear cell carcinoma (ccRCC) tissues and cells. USP7 removes ubiquitin modifications from HIF-2α, significantly inhibiting its proteasomal degradation. This leads to HIF-2α accumulation and the activation of downstream genes, such as EPO and vascular endothelial growth factor (VEGF), promoting tumor angiogenesis and growth. Experimental depletion of USP7 enhances HIF-2α degradation and effectively inhibits renal cancer progression, offering a novel therapeutic strategy (19).

### 1.2 Regulation of Hb by EPO

#### 1.2.1 Mechanism of EPO in erythropoiesis

**Figure 2:**
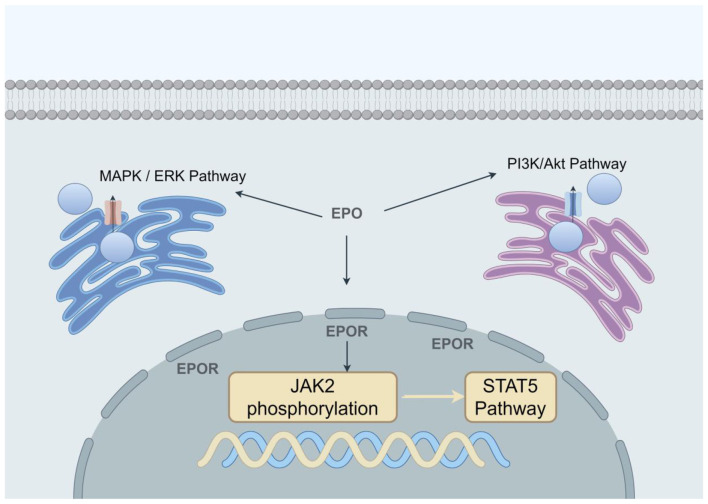
Mechanism of action of EPO in erythropoiesis Schematic diagram: Classical and non-classical pathways. This figure summarizes the four core signaling branches activated upon binding of EPO to its EPOR and their synergistic roles in erythropoiesis. The EPO/EPOR complex primarily transmits survival and differentiation signals through the JAK2-STAT5 pathway, drives cell cycle progression via the RAS-MAPK pathway, and regulates cellular metabolism and survival through the PI3K-AKT pathway. Additionally, calcium signaling pathways finely regulate late-stage hemoglobin synthesis during differentiation. These pathways do not operate in isolation but collaborate precisely in both time and space to ensure efficient proliferation of erythroid progenitor cells, prevent apoptosis, and ultimately facilitate differentiation into mature red blood cells.

EPO is a key regulator of erythropoiesis, exerting multifaceted effects on erythroid progenitor cells through various pathways involving signal transduction, iron metabolism, and modulation of the cellular microenvironment. The canonical pathway involves EPO binding to erythropoietin receptor (EPOR) on erythroid progenitors, triggering janus kinase 2 (JAK2) phosphorylation and subsequent activation of the signal transduction and activation of transcription factor 5 (STAT5) pathway. This promotes progenitor cell proliferation and differentiation while inhibiting apoptosis (20–22). The non-classical EPO pathway can also activate the mitogen-activated protein kinase/extracellular signal-regulated kinase (MAPK/ERK) and phosphoinositide 3-kinase/protein kinase B (PI3K/Akt) pathways (Figure 2): The MAPK/ERK pathway regulates cyclin D1, promoting cell cycle progression, while the PI3K/Akt pathway inhibits apoptosis by promoting the phosphorylation of FoxO3a, thereby enhancing the proliferation of erythroid progenitor cells (21).

EPO also activates the mitochondrial iron transporter-1, facilitating Fe^2^^+^ import into mitochondria. This activates heme-synthesizing enzymes, such as δ-aminolevulinic acid synthetase, thereby accelerating heme assembly (23).

Both epigenetic and microenvironmental factors regulate erythropoiesis. EPO can induce the recruitment of histone acetyl transferases (e.g., p300) to erythroid gene promoter regions, promoting chromatin relaxation (e.g., opening the β-globin gene cluster, *HBB*) and increasing transcription of Hb subunits (24, 25). Within the bone marrow niche, EPO synergizes with stem cell factor and interleukin-3 to maintain adhesion between erythroid progenitors and stromal cells, sustaining a microenvironment conducive to red blood cell maturation (26, 27) (Table 1).

**Table 1:** Major branches of the EPO signaling pathway and their roles in erythropoiesis.

**Signaling pathway**	**Key molecules**	**Biological function**	**Target genes/effects**
Janus kinase 2-signal transduction and activation of transcription factor 5 pathway (JK2-STAT5) (94–96)	JAK2, STAT5	Inhibits apoptosis, promotes differentiation	B-cell lymphoma-extralarge (BCL-XL), GATA-binding protein 1 (GATA-1)
Rat sarcoma virus-mitosin activated protein kinase pathway (RAS-MAPK) (21, 97, 98)	Growth factor receptor binding Protein 2 (Grb2), extracellular signal-regulated kinase (ERK)	Cell cycle progression	Cyclin D1, myeloma oncogene (c-Myc)
Phosphatidylinositol 3-kinase-protein kinase B pathway (PI3K-AKT) (99–101)	Protein kinase B (AKT), FOXO3a	Survival, metabolic reprogramming	Transferrin receptor (TfR), δ-Aminolevulinic Acid Synthase 2 (ALA2)
Ca^2+^ signaling (21, 102)	Phospholipase Cγ, activates nuclear factor of activated T cells	Fine-tunes differentiation	Heme synthesis enzymes

This table summarizes the four core signaling branches activated upon binding of EPO to its EPOR and their synergistic roles in erythropoiesis. The EPO/EPOR complex primarily transmits survival and differentiation signals through the JAK2-STAT5 pathway, drives cell cycle progression via the RAS-MAPK pathway, and regulates cellular metabolism and survival through the PI3K-AKT pathway. Additionally, calcium signaling pathways finely regulate late-stage hemoglobin synthesis during differentiation. These pathways do not operate in isolation but collaborate precisely in both time and space to ensure efficient proliferation of erythroid progenitor cells, prevent apoptosis, and ultimately facilitate differentiation into mature red blood cells.

#### 1.2.2 Dose-Response relationship between EPO and Hb

The EPO-Hb dose-response relationship is nonlinear. When Hb falls below 100 g/L, EPO levels increase inversely with Hb concentration, showing a significant negative correlation (28). When Hb is maintained between 100–150 g/L, a dynamic equilibrium exists. In pathological conditions, this regulation can become dysregulated (29). For example, in chronic mountain sickness (CMS) patients with Hb >200 g/L, EPO levels remain elevated, indicating a loss of normal feedback control (28). In renal anemia, absolute or relative EPO deficiency leads to Hb levels typically below 60–90 g/L, necessitating exogenous EPO supplementation or correction with hypoxia-inducible factor prolyl hydroxylase inhibitor (HIF-PHI). In cancer patients with anemia, tumor-induced microinflammation can blunt the EPO response (30). For these patients, treatment with erythropoiesis-stimulating agents (e.g., epoetin alfa) at Hb ≤ 100 g/L can correct anemia, with target levels generally maintained between 110–120 g/L (31, 32).

### 1.3 Physiological integration and feedback regulation of the HIF-2α-EPO–Hb axis

In summary, the HIF-2α-EPO–Hb axis constitutes a core physiological circuit that maintains oxygen homeostasis through precise feedback regulation. This axis follows a hierarchical “sensing-regulation-effect” logic: hypoxia signals initiate the response by stabilizing HIF-2α, a key transcription factor that activates EPO gene expression, converting hypoxia signals into hormonal commands. EPO then acts on the bone marrow to stimulate erythropoiesis and hemoglobin synthesis, thereby enhancing the blood's oxygen-carrying capacity. This process exhibits negative feedback regulation: when oxygen supply improves, the axis's activity diminishes, preventing excessive responses and maintaining dynamic equilibrium (33). Therefore, disruption at any point along this axis—such as the stability of HIF-2α, EPO transcription, or its downstream signaling pathways—can upset this delicate equilibrium, directly leading to disease. This provides a unified physiological framework for discussing the abnormalities observed in this axis under various pathological conditions.

## 2 Impact of hypoxia on HIF-2α expression

### 2.1 Differential expression of HIF-2α under acute vs. chronic hypoxia

During the early phase of acute hypoxia (minutes to hours), cells primarily mount rapid adaptive responses mediated by HIF-1α. A sudden drop in oxygen concentration rapidly inhibits PHD activity, leading to a swift accumulation of HIF-1α protein within minutes (5–30 min) (34) (Table 2). This activates downstream genes involved in glycolytic metabolic reprogramming, vasodilation, and cell survival. In acute hypoxia, HIF-1α mRNA is rapidly upregulated, and the protein accumulates quickly. In contrast, HIF-2α transcriptional activation and protein accumulation occur more slowly and to a lesser extent initially. HIF-2α also has a significantly longer half-life than HIF-1α, underpinning its role in sustained responses rather than rapid adaptation (34). As hypoxia persists (days to weeks), the HIF expression profile shifts to one dominated by HIF-2α. Under prolonged hypoxic conditions (e.g., high altitude or chronic disease states), HIF-1α protein levels begin to decline after approximately 24–48 h, whereas HIF-2α stability continues to increase, assuming the dominant role in the hypoxic response. This “relay” regulatory model allows the organism to deploy different functions at various stages: HIF-1α drives immediate survival responses during the acute phase, while HIF-2α facilitates long-term adaptation to chronic hypoxia.

**Table 2:** Core timeline of HIF subtype dynamics during hypoxia.

**Hypoxia duration**	**HIF-1α dynamics**	**HIF-2α dynamics**	**Dominant factor**	**References**
5–30 min	Rapid accumulation to detectable levels	Virtually no expression	HIF-1α	(34, 103)
4–8 h	Peak (acute response peak)	Slow increase	HIF-1α	(104, 105)
12–24 h	Significant degradation to basal levels	Stable accumulation	Transition period	(34, 106)
48 h	Decreased to low levels	Expression surpasses HIF-1α	HIF-2α	(9, 107)
≥72 h	Maintains low levels or fluctuates	Sustained high expression (long-term adaptation)	HIF-2α	(108, 109)

This table summarizes the temporal transition in HIF-1α and HIF-2α expression dynamics during the continuum from acute to chronic hypoxia. In the initial phase of hypoxia (within hours), HIF-1α rapidly accumulates and dominates the transcription of early stress genes, executing rapid compensation; As hypoxia persists (24–48 h), HIF-1α gradually degrades while HIF-2α expression steadily increases and ultimately becomes dominant, regulating genes associated with long-term adaptation such as EPO. This “relay” regulation from HIF-1α to HIF-2α reflects the cell's sophisticated temporal control mechanism for transitioning hypoxia signaling from acute response to long-term adaptation.

The natural hypoxic gradient associated with increasing altitude has been used to investigate changes in HIF-2α expression at different oxygen partial pressures, revealing human compensatory mechanisms. At moderate altitudes (1,500–2,500 m), representing mild hypoxic stress, HIF-2α levels increase gradually. Under simulated hypoxic conditions (cold stress at 16 °C), HIF-2α is specifically activated in adipocytes. This promotes ceramide catabolism by inducing the expression of alkaline ceramidase 2, thereby inhibiting the development of atherosclerosis (35, 36). This may be a key mechanism for the cardiovascular protective effects observed under moderate hypoxia. At higher altitudes (>3,000 m, equivalent to <14% O_2_), significant hypoxia triggers a more pronounced increase in HIF-2α expression, which then plays a crucial regulatory role. At this stage, HIF-2α enhances erythropoiesis and blood oxygen-carrying capacity, improves mitochondrial respiratory function, and activates antioxidant systems to mitigate oxidative stress damage (37–39). At extreme altitudes (>5,500 m, partial pressure of oxygen <11%), HIF-2α levels may plateau or even decrease, preventing potential harm from excessive activation (40, 41) (Table 3). This adaptive adjustment point likely aids survival in extreme environments.

**Table 3:** Comparative expression profiles of HIF subtypes in acute and chronic hypoxia.

**Characteristic**	**Acute hypoxia (minutes–hours)**	**Acute hypoxia (minutes–hours)**	**Key evidence sources**
Dominant HIF subtype	Primarily HIF-1α	Primarily HIF-2α	(33, 110, 111)
Stabilization rate	Rapid (minutes)	Slow (hours–days)	(33, 111, 112)
Primary biological function	Glycolysis activation, acute inflammatory response	Promotes erythropoiesis, vascular remodeling	(60, 64, 72)
Degradation kinetics	Rapid degradation upon reoxygenation	Steady decline, partial tissue specificity	(33, 111, 112)
Typical downstream pathways	Glucose Transporter1 (GLUT1), Glycerophosphate Kinase 1 (PGK1),VEGF, Acute reaction	EPO, antioxidant enzymes, Mitochondrial respiration regulation	(33, 60, 111)

This table summarizes the temporal switching mechanism of HIF-1α and HIF-2α in cells responding to hypoxia: During acute hypoxia, HIF-1α is rapidly induced and activated, primarily initiating immediate metabolic adaptations such as glycolysis and inflammatory responses. As hypoxia persists, HIF-2α gradually assumes dominance, shifting to regulate long-term adaptive physiological processes like erythropoiesis and vascular remodeling. This “relay” model demonstrates how the body achieves a dynamic response to hypoxia signals—transitioning from rapid stress to sustained adaptation—through the differential activation of distinct HIF subtypes.

### 2.2 Tissue- and cell-type-specific variations and physiological functions of HIF-2α

#### (1) Renal tissue

- **Renal glomerular endothelial cells:** Under hypoxia, functional upregulation of HIF-2α reduces the expression of tight junction proteins occludin and tight junction protein-1, decreases trans-epithelial electrical resistance, increases cellular permeability, and promotes proteinuria. Inhibition of HIF-2α reverses these effects (42, 43).
- **Renal interstitial cells:** Chronic hypoxia continuously stimulates HIF-2α to induce EPO expression (44).

#### (2) Hepatic tissue

- **Hepatocyte metabolic regulation:** Simulating a 5,000 m altitude hypoxic environment increases HIF-2α expression in rat liver. Furthermore, by reducing the expression of key gluconeogenic enzymes—glucose-6-phosphatase catalytic subunit and phosphoenolpyruvate carboxylase kinase—in rat liver, blood glucose levels are lowered, thereby maintaining the body's energy homeostasis (45, 46).
- **Iron metabolism regulation:** HIF-2α can also directly regulate duodenal iron transporters such as DMT1 and FPN (Ferri-transporter). In iron-deficiency anemia models, it promotes mRNA and protein expression of DMT1 and FPN in the mouse intestine, enhancing iron absorption (17, 47, 48). In contrast, in β-thalassemia, aberrant HIF-2α activation downregulates the expression of genes and proteins like ferritin, μ-ferritin transporter (μ-FPN), and uremic toxin receptor (u-POR), leading to pathological iron overload (49, 50).

#### (3) Lung and brain tissues

In the lung, HIF-2α contributes to the pathophysiology of pulmonary arterial hypertension. In the central nervous system, HIF-2α is expressed in cells like astrocytes and may influence neural function and adaptation by regulating related factors.

#### (4) Endothelial cells

During systemic angiogenesis, chronic hypoxia promotes collateral circulation formation via the HIF-2α-VEGF axis (51).

## 3 Dysregulation of the HIF-2α-EPO–Hb Axis in disease

### 3.1 Chronic systemic diseases

In CKD, renal tissue fibrosis disrupts interstitial fibroblast function, impairs HIF-2α activation, and leads to insufficient EPO secretion and renal anemia. Uncorrected anemia further exacerbates systemic organ hypoxia, creating a vicious cycle. Additionally, uremic toxins (e.g., indoxyl sulfate) accumulated in CKD patients can suppress HIF-2α transcriptional activity, worsening EPO synthesis (52, 53). The cardiorenal anemia syndrome further complicates this interplay: cardiac insufficiency reduces renal perfusion, while renal anemia increases cardiac workload, elevating the risk of both heart and renal failure (54–56). In COPD, persistent hypoxemia would normally stimulate EPO production. However, concomitant systemic inflammation inhibits erythropoiesis, leading to relative EPO deficiency. The literature reports a U-shaped relationship between Hb levels and prognosis in COPD: both mild anemia (Hb 100–120 g/L) and severe polycythemia (Hb >160 g/L) are associated with a higher risk of hospitalization. This underscores the need for dynamic monitoring and multidimensional interventions (e.g., anti-inflammatory therapy, nutritional support, renal function management) to maintain Hb within an optimal range (57–59).

### 3.2 Abnormal expression in tumors

In tumors such as ccRCC and hepatocellular carcinoma (HCC), mutations in genes like the von Hippel-Lindau (VHL) gene, combined with the hypoxic microenvironment within the tumor, lead to the persistent activation and loss of normal regulation of the HIF-2α-EPO-Hb axis. This abnormal activation not only stimulates erythropoiesis to promote tumor proliferation but also directly serves as a key driver of further malignant progression.

In ccRCC, the dysregulation of the HIF-2α-EPO–Hb axis represents a core molecular driver of the disease (33). This stems from frequent inactivation of the VHL tumor suppressor gene, preventing normal degradation of HIF-2α protein even under normoxic conditions and establishing a persistent state of abnormal activation (1, 60). This dysregulated axis drives tumor progression through multiple pathways: sustained HIF-2α activation massively upregulates key factors like VEGF and platelet-derived growth factor (PDGF), promoting chaotic and non-beneficial angiogenesis within tumor tissues. However, these dysfunctional neo-vessels exacerbate hypoxia within the tumor, creating a self-reinforcing positive feedback loop that further stabilizes HIF-2α activity (1, 61). Meanwhile, EPO—a primary downstream target of HIF-2α–undergoes abnormal transcriptional activation, frequently resulting in paraneoplastic erythrocytosis in patients. Elevated EPO can influence tumor cells via its receptor, the EPOR, potentially promoting their proliferation and survival. Furthermore, it increases hemoglobin levels, thereby altering oxygen status and blood rheology within the tumor microenvironment (12, 61). Precisely due to this deep understanding of the axis, HIF-2α-specific inhibitors (such as Belzutifan) have become a primary therapeutic strategy for ccRCC. By disrupting this pathogenic axis, they simultaneously inhibit tumor growth, abnormal angiogenesis, and excessive EPO production (60).

In HCC, abnormal expression and regulation of the HIF-2α-EPO-Hb axis are central to tumorigenesis, progression, and treatment sensitivity. HIF-2α exhibits abnormal stabilization and activation driven by multiple factors, including the hypoxic tumor microenvironment, mutations in the hypoxia-inducible factor 2α gene (*HIF2A*), and the cyclooxygenase-2/prostaglandin E2 (COX-2/PGE2) axis (33). This persistently activated HIF-2α enhances VEGF, significantly promoting HCC cell proliferation, invasion, metastasis, and angiogenesis, indicating poor prognosis (1). Concurrently, HIF-2α participates in tumor metabolic reprogramming and influences cellular proliferation through interactions with HIF-1α (1, 2). HIF-2α is also a key target for sorafenib resistance, and its inhibition enhances treatment sensitivity (60). EPO serves as a downstream effector of HIF-2α, with its gene transcription directly regulated by HIF-2α. HIF-2α binds to the EPO gene enhancer, driving EPO overexpression (62). Although EPO primarily regulates erythropoiesis, elevated levels of EPO and its EPOR in HCC correlate closely with tumor growth and poor prognosis. They also stimulate VEGF expression via the HIF-1 signaling pathway, thereby enhancing angiogenesis. Patients with HCC and polycythemia vera often harbor germline mutations in *HIF2A*, conferring gain-of-function to HIF-2α and abnormally promoting EPO production, leading to secondary polycythemia. EPO production is also influenced by iron metabolism, indirectly affecting erythropoiesis and tumor progression through interactions with ferritin (63). Hb, as the primary oxygen-carrying protein, directly reflects blood's oxygen-carrying capacity. Erythrocytosis induced by HIF-2α mutations and elevated EPO leads to abnormal Hb elevation, altering blood viscosity and consequently affecting hemodynamics and oxygen supply within the tumor microenvironment. Hypoxia in the HCC tumor microenvironment promotes tumor invasion, metastasis, and resistance to treatment (64). Hb's oxygen-carrying function is crucial for maintaining tumor oxygenation, and the associated polycythemia may represent an adaptive response aimed at optimizing oxygen supply within the tumor region. In summary, the HIF-2α-EPO-Hb axis plays a complex and critical role in HCC proliferation, invasion, metastasis, angiogenesis, metabolic reprogramming, and treatment resistance. Deepening our understanding of the dysregulated mechanisms within this axis may offer new diagnostic biomarkers and therapeutic targets for HCC.

### 3.3 Metabolic disorders

Although direct evidence for the HIF-2α-EPO–Hb axis in obesity and non-alcoholic fatty liver disease (NAFLD) remains relatively limited, its potential impact warrants further investigation given the axis's central role in hypoxia response and metabolic regulation. Obesity and NAFLD states are frequently accompanied by chronic low-grade inflammation and tissue fibrosis, potentially causing local microcirculatory impairment and functional hypoxia in the liver. This creates a potential microenvironmental basis for the abnormal activation of HIF-2α (1). Speculative studies suggest that dysregulation of this axis may contribute to disease progression through multiple mechanisms: on one hand, activated HIF-2α may alter hepatic metabolism, promoting increased lipid accumulation in the liver and suppressing thermogenesis in adipose tissue, thereby exacerbating metabolic disorders; on the other hand, abnormal production of its downstream target EPO may interact with iron metabolism disorders, affecting systemic energy balance. Although the direct driving role of this axis in NAFLD progression remains unconfirmed, elucidating its mechanisms offers new perspectives for explaining metabolic disease heterogeneity and developing targeted interventions. Future studies should validate the specific regulatory networks and pathological significance of this axis in clinical cohorts and experimental models.

### 3.4 High-altitude diseases

CMS is characterized by excessive hemoglobin elevation (Hb >210 g/L in males, >190 g/L in females), with its core mechanism rooted in the sustained activation of the HIF-2α signaling pathway. Molecular studies reveal that HIF-2α, EPO, and VEGF positivity rates in bone marrow tissues of CMS patients reach 88.24%, 88.24%, and 85.29%, respectively, significantly higher than those in the control group (66.67%, 50.00%, 56.67%) (65). This abnormal activation leads to hyperplasia of the erythroid lineage in the bone marrow, with EPO stimulating erythroid precursor proliferation through autocrine/paracrine pathways (66). In patients with CMS, localized activation of the HIF-2α/EPO pathway in bone marrow significantly increases microvascular density, providing structural support for erythropoiesis (65). Hypoxia induces VEGF expression by upregulating HIF-1α, promoting vascular endothelial cell proliferation, migration, and neovascularization, thereby accelerating bone marrow vascular remodeling (67, 68). Iron metabolism disorders: Despite elevated Hb levels, approximately 30% of CMS patients exhibit functional iron deficiency, indicating inadequate iron supply to meet hematopoietic demands (65). Genetic studies reveal that the Tibetan population harbors positive-selection mutations in the endothelial PAS domain-containing protein 1 (EPAS1, the HIF-2α-encoding gene) exhibits positive selection mutations such as aspartic acid replaced by tyrosine. This results in lower HIF-2α activity at equivalent altitudes compared to lowland populations, demonstrating a unique molecular adaptation mechanism to high-altitude hypoxia. This adaptation prevents increased blood viscosity and cardiovascular risks associated with polycythemia (69–71).

In the hypoxic environment of high altitudes, abnormal expression and regulation of the HIF-2α-EPO-Hb axis are critical for organismal adaptation and disease progression. During hypoxia, HIF-2α stabilizes and accumulates due to suppressed prolyl hydroxylase activity, subsequently activating EPO expression. This promotes increased erythrocytosis and elevated Hb levels to enhance oxygen transport capacity. While this adaptive response is generally effective, excessive or prolonged HIF-2α activation leads to dysregulation, resulting in secondary polycythemia. This condition increases blood viscosity, elevates cardiac workload and thrombotic risk, and constitutes a hallmark feature of CMS (38, 72, 73). Upon activation, HIF-2α also induces metabolic reprogramming to sustain cellular energy supply and may exacerbate inflammatory responses (74). Consequently, HIF-2α, EPO, and Hb levels serve as potential diagnostic markers for both acute and chronic mountain sickness. Therapeutically, HIF-2α inhibitors have demonstrated efficacy in renal clear cell carcinoma, suggesting potential applications for improving high-altitude erythrocytosis. Similarly, modulating the EPO signaling pathway to prevent excessive erythropoiesis holds therapeutic promise (60). Deepening our understanding of the regulatory mechanisms within this axis will provide crucial insights for early diagnosis, risk assessment, and intervention strategy development for high-altitude sickness.

To clearly contrast and highlight the specific dysregulation patterns of the HIF-2α-EPO–Hb axis across the aforementioned disease categories, its core characteristics are summarized in (Table 4).

**Table 4:** Specific dysregulation characteristics of the HIF-2α-EPO–Hb axis.

**Disease context**	**Core dysregulation**	**Key molecular features/drivers**	**Functional consequences**	**Clinical/therapeutic implications**
Chronic Hypoxic Diseases (e.g., CKD Anemia)	Relative or absolute HIF-2α deficiency reduces EPO production	Renal interstitial fibrosis/inflammation inhibits HIF-2α transcription; PHD enzymes continuously hydroxylate HIF-2α, leading to degradation	Tissue hypoxia-anemia vicious cycle exacerbates organ damage; increased cardiovascular complication risk	Activate HIF-2α axis: HIF-PHIs, stabilize HIF-2α to promote endogenous EPO production, representing breakthrough therapies for renal anemia correction (33, 110, 113–115)
High-altitude-related diseases (e.g., CMS)	HIF-2α overactivation leads to excessive EPO production	Genetic adaptation variations (e.g., EPAS1 mutations); persistent environmental hypoxia stabilizes HIF-2α	Excessive erythropoiesis causes polycythemia and blood hyperviscosity, increasing cardiovascular risk	Inhibition or modulation of the HIF-2α axis: Strategies for safely downregulating HIF-2α/EPO require exploration; investigate the translational potential of adaptive mutations like EPAS1 (33, 60, 112, 116)
Tumors (e.g., ccRCC)	Persistent, dysregulated activation of HIF-2α	Loss-of-function VHL mutations; tumor microenvironment hypoxia; HIF-2α dimerization with HIF-1β	Oncogenic Effects: Drives proliferation, angiogenesis, immune evasion, and treatment resistance	Inhibition of the HIF-2α axis: Belzutifan, a HIF-2α-specific antagonist, emerges as a novel targeted therapy for ccRCC by blocking HIF-2α dimerization; Explore strategies like combination immunotherapy to enhance efficacy (2, 33, 60, 116)

This table compares the dysregulation characteristics of the HIF-2α-EPO–Hb axis in three typical disease contexts. In chronic hypoxic diseases (such as CKD anemia), this axis exhibits hypofunction and requires activation via HIF-PHIs; In high-altitude-related diseases, the axis exhibits excessive activation, necessitating therapeutic strategies focused on downregulation; whereas in tumors (e.g., ccRCC), the axis demonstrates persistent, uncontrolled activation, requiring inhibition via HIF-2α antagonists. These three distinct dysregulation patterns directly correspond to three fundamentally opposing therapeutic rationales, underscoring the clinical significance of precisely targeting this axis.

## 4 Applications of HIF modulators

### 4.1 Applications of EPO and its analogs

Recombinant human EPO (rHuEPO) and long-acting analogs (e.g., darbepoetin α) are foundational therapies for renal anemia. While effective in raising Hb levels, their use is associated with several issues:

**EPO resistance:** Approximately 10% of dialysis patients show no clinical improvement even with high EPO doses (>300 IU/kg/week), a phenomenon termed EPO resistance (75–77), often due to iron deficiency, microinflammation, or immune-mediated pure red cell aplasia.
**Significant Hb fluctuations:** The short half-life of rHuEPO (4–13 h) can lead to large Hb swings (30–40 g/L), potentially increasing cardiovascular risk (78–80).
**Increased hypertension incidence:** EPO may induce hypertension via vasoconstrictive effects (81). To address these limitations, novel erythropoietin receptor agonists, such as peginesatide (a pegylated peptide), have been developed. Its modified structure prolongs its half-life and avoids cross-reactivity with EPO antibodies. Compared with rHuEPO, it allows less frequent administration (e.g., monthly) and reduces Hb fluctuations (82, 83).

### 4.2 HIF pathway modulators

**Figure 3:**
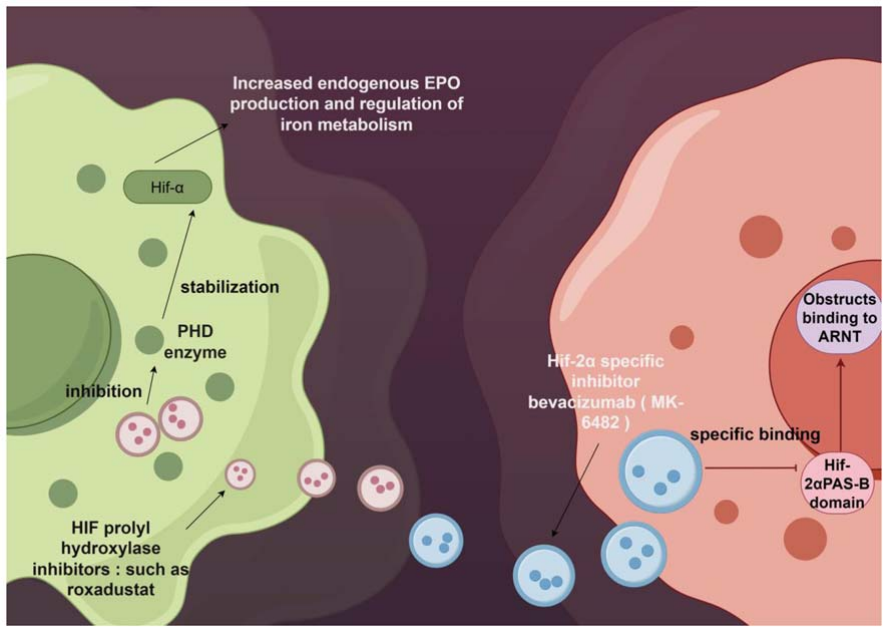
Mechanism Diagram of HIF Pathway Regulators. Summarizes two regulatory strategies targeting HIF-2α. The left panel indicates that HIF-PH inhibitors (e.g., roxadustat) stabilize HIF-α subunits by inhibiting PHD activity, thereby promoting endogenous EPO production and improving iron metabolism. This approach can be used to treat conditions such as renal anemia. The right panel depicts HIF-2α-specific inhibitors (e.g., Belzutifan/MK-6482), which directly block HIF-2α binding to ARNT, suppressing its transcriptional activity. These are employed to treat tumors dependent on this pathway, such as renal clear cell carcinoma. Together, they represent distinct therapeutic approaches—activation versus inhibition—of this axis. This figure was created using the Figdraw platform.

Drugs targeting the HIF pathway have opened new avenues for treating anemia and tumors. HIF-PHIs: Agents like roxadustat inhibit PHD enzymes, stabilize HIF-α, increase endogenous EPO production, and modulate iron metabolism (84, 85). HIF-2α-Specific Inhibitors: Belzutifan (MK-6,482) selectively binds to the PAS-B domain of HIF-2α, preventing its dimerization with ARNT (Figure 3), thereby blocking HIF-2α heterodimer formation. In ccRCC patients, it has achieved an objective response rate of 22% and a complete response rate of 4%, with favorable tolerability (9, 86, 87). As an oral HIF-2α inhibitor, Belzutifan selectively blocks HIF-2α activity, suppressing the expression of downstream oncogenes (e.g., VEGF, PDGF), inhibiting tumor angiogenesis and proliferation. In VHL syndrome-associated renal cell carcinoma, treatment with Belzutifan has led to tumor shrinkage exceeding 30% in some cases (9, 88, 89).

## 5 Limitations and future perspectives

### 5.1 Limitations of current research

Current research on the HIF-2α-EPO–Hb axis faces several challenges:

1. Incomplete molecular understanding: Certain molecular mechanisms require further elucidation. For example, the detailed process of USP7-mediated de-ubiquitination in renal cancer and the comprehensive interaction network between non-coding RNAs and HIF-2α remain to be fully mapped.
2. Limited understanding of disease-specific regulation: The reasons for tissue-specific regulation—such as why the kidney primarily uses HIF-2α for EPO regulation while the liver employs both HIF-1α and HIF-2α, and why different tissues exhibit varied responses to HIF-2α activation—are not fully understood.
3. Drug selectivity and safety issues: Current HIF-PHIs stabilize both HIF-1α and HIF-2α, potentially carrying tumorigenic risks. While Belzutifan exhibits greater selectivity, acquired mutations (e.g., gatekeeper mutation) can confer resistance. For anemia treatment, the long-term safety of HIF-PHIs (particularly regarding tumor risk) requires validation in larger patient cohorts.
4. Lack of targeted therapies for specific conditions: For high-altitude illnesses like CMS, no targeted molecular therapies are currently available. Current treatment still relies on traditional phlebotomy, which is suboptimal and associated with poor patient compliance.

### 5.2 Future outlook and translational challenges

To facilitate the translation of HIF-2α-EPO–Hb axis-related therapies into precise and safe clinical applications, future research should prioritize three key areas. First, the development of tissue- and cell-specific regulatory strategies is essential. This may involve targeting kidney- or liver-specific proteins, such as sodium-glucose cotransporter 2 (SGLT2) or asialoglycoprotein receptor 1 (ASGR1), for targeted delivery (90–92), or designing small-molecule mimetics tailored to specific genetic backgrounds (72), such as the HIF-2α mutation found in the Tibetan population. Additionally, technologies such as clustered regularly interspaced short palindromic repeats/nuclease-deficient Cas9 (CRISPR/dCas9) can be utilized to precisely regulate endogenous EPO gene expression, thereby minimizing adverse effects associated with excessive drug concentrations (93). Building on these efforts, the second priority is to intensify long-term monitoring of drug safety and actively explore novel synergistic treatment approaches. This includes a comprehensive assessment of potential risks related to the expanded and prolonged use of HIF-PHIs and HIF-2α inhibitors, such as tumor promotion or vascular abnormalities, as well as the investigation of combination therapies to enhance efficacy and overcome resistance. For example, co-administration of HIF-PHIs with immune checkpoint inhibitors in ccRCC or with iron regulator blockers for anemia treatment may be beneficial. Finally, establishing a robust biomarker system to predict treatment efficacy and monitor therapeutic responses is critical. To this end, integrating multi-omics data and employing advanced technologies such as single-cell sequencing and spatial transcriptomics will enable the identification of reliable biomarkers to guide personalized therapy. By implementing these strategies, it is expected that the development of innovative therapies will be driven in the coming decade, ultimately improving treatment outcomes for anemia, tumors, and high-altitude hypoxia-related diseases.

## 6 Conclusion

The HIF-2α-EPO-Hb axis maintains homeostasis via a sensitive negative feedback loop. Hypoxia stabilizes HIF-2α, increasing EPO expression, which stimulates hematopoiesis and hemoglobin production to enhance oxygen delivery. This regulation is conditional, varying with hypoxia duration and tissue type. Axis dysregulation creates distinct disease patterns: hypoactivity in conditions like renal anemia, and compensatory or persistent hyperactivity in high-altitude sickness and renal clear cell carcinoma, respectively. These patterns dictate therapies to either enhance or suppress axis activity. Translational advances, like HIF prolyl hydroxylase inhibitors and HIF-2α antagonists, validate its therapeutic relevance. Remaining challenges include achieving tissue-selective regulation, ensuring long-term safety, and developing biomarkers for personalized therapy. Thus, the HIF-2α-EPO-Hb axis critically links oxygen sensing to systemic response, and studying its disease-specific alterations will advance the understanding and treatment of hypoxia-related pathophysiology.
